# Association of Stress With Cognitive Function Among Older Black and White US Adults

**DOI:** 10.1001/jamanetworkopen.2023.1860

**Published:** 2023-03-07

**Authors:** Ambar Kulshreshtha, Alvaro Alonso, Leslie A. McClure, Ihab Hajjar, Jennifer J. Manly, Suzanne Judd

**Affiliations:** 1 Department of Epidemiology, Rollins School of Public Health, Emory University, Atlanta, Georgia; 2 Department of Family and Preventive Medicine, Emory University School of Medicine, Atlanta, Georgia; 3 Department of Epidemiology and Biostatistics, Dornsife School of Public Health, Drexel University, Philadelphia, Pennsylvania; 4 Department of Neurology, University of Texas Southwestern Medical Center, Dallas; 5 Taub Institute for Research on Alzheimer’s Disease and the Aging Brain, Columbia University, New York; 6 Department of Biostatistics, University of Alabama at Birmingham, Birmingham; 7 Department of Epidemiology, University of Alabama at Birmingham, Birmingham

## Abstract

**Importance:**

Perceived stress can have long-term physiological and psychological consequences and has shown to be a modifiable risk factor for Alzheimer disease and related dementias.

**Objective:**

To investigate the association between perceived stress and cognitive impairment in a large cohort study of Black and White participants aged 45 years or older.

**Design, Setting, and Participants:**

The Reasons for Geographic and Racial Differences in Stroke (REGARDS) study is a national population-based cohort of 30 239 Black and White participants aged 45 years or older, sampled from the US population. Participants were recruited from 2003 to 2007, with ongoing annual follow-up. Data were collected by telephone, self-administered questionnaires, and an in-home examination. Statistical analysis was performed from May 2021 to March 2022.

**Exposures:**

Perceived stress was measured using the 4-item version of the Cohen Perceived Stress Scale. It was assessed at the baseline visit and during 1 follow-up visit.

**Main Outcomes and Measures:**

Cognitive function was assessed with the Six-Item Screener (SIS); participants with a score below 5 were considered to have cognitive impairment. Incident cognitive impairment was defined as a shift from intact cognition (SIS score >4) at the first assessment to impaired cognition (SIS score ≤4) at the latest available assessment.

**Results:**

The final analytical sample included 24 448 participants (14 646 women [59.9%]; median age, 64 years [range, 45-98 years]; 10 177 Black participants [41.6%] and 14 271 White participants [58.4%]). A total of 5589 participants (22.9%) reported elevated levels of stress. Elevated levels of perceived stress (dichotomized as low stress vs elevated stress) were associated with 1.37 times higher odds of poor cognition after adjustment for sociodemographic variables, cardiovascular risk factors, and depression (adjusted odds ratio [AOR], 1.37; 95% CI, 1.22-1.53). The association of the change in the Perceived Stress Scale score with incident cognitive impairment was significant in both the unadjusted model (OR, 1.62; 95% CI, 1.46-1.80) and after adjustment for sociodemographic variables, cardiovascular risk factors, and depression (AOR, 1.39; 95% CI, 1.22-1.58). There was no interaction with age, race, and sex.

**Conclusions and Relevance:**

This study suggests that there is an independent association between perceived stress and both prevalent and incident cognitive impairment. The findings suggest the need for regular screening and targeted interventions for stress among older adults.

## Key Points

**Question:**

Is there an association between perceived stress and cognitive performance?

**Findings:**

This cohort study of 24 448 Black and White participants aged 45 years or older found an independent association between perceived stress and both prevalent and incident cognitive impairment.

**Meaning:**

This study suggests that there may be a need for screening for stress among high-risk older adults when they present in primary care.

## Teaser

This cohort study investigates the association between perceived stress and cognitive impairment in a large cohort of Black and White participants aged 45 years or older.

## Introduction

Studies have projected that a 10% to 25% reduction in modifiable risk factors, including behavioral factors, could prevent 1.3 million cases of Alzheimer disease globally.^1,2^ Perceived stress is defined as a consequence of events or demands that exceed an individual’s professed ability to cope.^3,4,5^ Perceived stress can have long-term physiological and psychological consequences and has been shown to be a modifiable risk factor for mild cognitive impairment and Alzheimer disease.^6,7^ Perceived stress among adults is associated with hormonal and inflammatory indicators of accelerated aging as well as excess risk of cardiovascular and stroke morbidity and mortality.^8,9,10,11^ It has also been associated with sleep problems and poor immunologic function.^12^ Studies have shown that the high prevalence of dementia in racial and ethnic minority groups, such as Black populations, may be partly attributable to high levels of stress associated with low socioeconomic status and discrimination throughout the life course.^13^ Perceived stress, especially in racial and ethnic minority groups, can directly affect cognition and also plays a role in worsening of unhealthy behaviors, such as smoking, physical inactivity, and reduced medication compliance.^14,15^ Despite the racial and ethnic disparities in dementia, few longitudinal studies with a diverse population have investigated the association between stress and cognitive impairment, to our knowledge.^16^ More studies are needed to rigorously test the association of chronic stress with cognitive decline at different points in the life cycle and in racially and ethnically diverse groups. Understanding the social and behavioral complexities associated with stress and unhealthy behaviors by race and ethnicity can help point toward interventions to prevent the progression of cognitive impairment.

We examined the association between perceived stress and prevalent and incident cognitive impairment (ICI) in the Reasons for Geographic and Racial Differences in Stroke (REGARDS) study, a large cohort study comprising Black and White participants. We also explored whether race, sex, and age modify the association between perceived stress and cognition. We hypothesized that higher levels of perceived stress would be associated with a higher risk of cognitive impairment and that this association would not be modified by race, sex, and age.

## Methods

The REGARDS study is a population-based investigation of stroke incidence and cognitive function among Black non-Hispanic and White non-Hispanic US adults aged 45 years or older.^17^ The study was designed to sample an equal proportion of women and men, but to oversample Black participants as well as people living in the southeastern US states where rates of stroke are highest in the US (commonly referred to as the “stroke belt” [North Carolina, South Carolina, Georgia, Tennessee, Mississippi, Alabama, Louisiana, and Arkansas] or the “stroke buckle” [coastal plains of North Carolina, South Carolina, and Georgia that have a higher stroke mortality rate than the remainder of the stroke belt]).^18^ The REGARDS sample was selected from a commercially available nationwide list purchased through Genesys Inc, stratified to reflect the specific age-race-sex-geographic strata. Sample listings were purchased in batches of 50 000 households to ensure the most current telephone numbers and addresses. Criteria for inclusion in the sample included having a name, telephone number, and address in the Genesys database. Overall, 30 239 participants were enrolled between 2003 and 2007, with ongoing annual follow-up. Potential participants were recruited through a mailing, followed by telephone calls. In this stratified random sample, 55.0% (16 632 of 30 239) were women, 41.4% (12 514 of 30 239) were Black, and 55.0% (16 625 of 30 239) were recruited from the southeastern US. In this analysis, we included REGARDS participants with data on cognition, perceived stress, physical activity, alcohol use, cigarette smoking, diabetes, hypertension, depressive symptoms, anxiety, and body mass index. From our original sample of 30 239 participants, we excluded individuals with anomalous data (n = 56), history of stroke (n = 1980), no baseline or missing Six-Item Screener (SIS) score (n = 490), coronary heart disease (n = 955), and severe SIS score or dementia (n = 2310), resulting in our final participant size of 24 448. The REGARDS study protocol was approved by the institutional review boards at the participating institutions (University of Alabama at Birmingham, University of Vermont, Wake Forest University, and Alabama Neurological Institute Inc), and all participants provided written informed consent. This study followed the Strengthening the Reporting of Observational Studies in Epidemiology (STROBE) reporting guideline.^19^

### Data Collection

Sociodemographic and clinical data were collected at baseline through a telephone interview, an in-home examination, and self-administered questionnaires left in the home. The in-home visit was scheduled after the telephone interview. Trained interviewers conducted computer-assisted telephone interviews to obtain information on participants’ demographic characteristics, cigarette smoking, physical activity, and use of medications. Trained health professionals conducted in-home visits that included a physical examination and collection of fasting blood samples, which were shipped overnight for analysis and storage to the University of Vermont.

Participants self-reported age, race, sex, household income, educational level, smoking status, alcohol use, and physical activity. Smoking status was categorized as never smokers, current smokers, or former smokers (smoked at least 100 cigarettes in a lifetime). Current alcohol use was assessed with 2 questions, and participants’ consumption was categorized as heavy (>14 drinks per week for men or >7 drinks per week for women), moderate (1-14 drinks per week for men or 1-7 drinks per week for women), or none per National Institute on Alcohol Abuse and Alcoholism guidelines.^20^ Physical activity was assessed with a single item (how often per week do you exercise enough to work up a sweat), and participants were dichotomized as engaging in no activity or any activity. Body mass index was calculated as weight in kilograms divided by height in meters squared. Blood pressure was measured using a sphygmomanometer after the participant rested for 5 minutes. During the in-home visits, information regarding current medication use for hypertension, diabetes, dyslipidemia, or depression was assessed via medication inventory review. Depressive symptoms were assessed using the abbreviated version of the Center for Epidemiologic Studies Depression Scale questionnaire, which has been previously validated and demonstrated high correlation with the original longer version (*r* = 0.87).^21^ Scores of 4 or more were indicative of elevated levels of depressive symptoms. Fasting blood samples were obtained and assayed for total cholesterol, high-density lipoprotein cholesterol, and glucose levels. Diabetes was defined as a fasting blood glucose level of more than 125 mg/dL (to convert to millimoles per liter, multiply by 0.0555) or self-reported history of diabetes or diabetes medication use.

### Exposure: Perceived Stress

The Cohen Perceived Stress Scale (PSS)^22^ is a widely used validated index of psychological stress that is associated with a broad range of outcomes and has a high 2-year stability. Perceived stress in the REGARDS study is measured during the baseline computer-assisted telephone interview with the 4-item version of the Cohen PSS. Scores on this measure range from 0 to 16. When examining perceived stress from a dichotomous perspective (ie, no stress or elevated stress), a PSS score greater than 5 (upper tertile of the scores reported by all participants in the REGARDS study) is used to classify participants as reporting elevated stress.^4,23,24^ When using a multicategorical approach, perceived stress categories were defined using PSS score tertiles from the current sample: low (0-1), moderate (2-4), or high (≥5). Perceived stress was measured at baseline and during 1 visit after 11 years of follow-up of REGARDS study participants.

### Outcome: Cognition

REGARDS study participants were administered the SIS during the baseline telephone interview beginning in 2003; the SIS has been administered annually. The SIS is a brief measure of global cognitive status that assesses recall of 3 words and orientation to year, month, and day of the week (score range, 0-6).^25^ The SIS can be measured both in person or using telephone administration and is derived from the widely used Mini-Mental State Examination.^26^ We defined cognitive impairment as scoring below 5 points on the SIS based on validity studies in clinical and community samples demonstrating an association of the SIS score with clinical diagnoses of mild cognitive impairment.^27,28^ We defined ICI as a shift from intact cognition (score of 5 or 6 correct on the SIS) at the first assessment to impaired cognition (score of ≤4 on the SIS) at the latest available assessment.^29^ Prior studies using the SIS established its utility in detecting impaired cognition.^30^ A score of 4 or less has a sensitivity of 74.2% to 84.0% and a specificity of 80.2% to 85.3% for a diagnosis of cognitive impairment based on evaluation in community and clinical samples.^25^

### Statistical Analysis

Statistical analysis was performed from May 2021 to March 2022. Characteristics of the study participants were described using mean (SD) values. We used *t* tests and χ^2^ tests of association for unadjusted comparisons between those with and those without elevated perceived stress. For our primary analyses, we assessed the PSS score as a continuous variable and also as a categorical variable (PSS score >5). We first used logistic regression to estimate odds ratios (ORs) of cognitive impairment (based on the SIS score) for low vs high perceived stress. Odds ratios were adjusted sequentially in different models including (1) age, sex, educational level, race, and household income; (2) cardiovascular disease (CVD) risk factors including hypertension, diabetes, and dyslipidemia; (3) lifestyle factors including physical inactivity, obesity, and smoking; and (4) depression. We also explored race, sex, and age as potential effect modifiers by including interaction terms in the fully adjusted model.^31^ REGARDS study participants are followed up at fixed intervals when cognition tests are administered. Thus, time-to-event methods will be difficult to apply for these analyses because we have an interval censoring problem. For the follow-up analyses, we adjusted for the time to the first ICI to estimate the OR for the association between change of perceived stress levels and ICI. Model validation was done by creating a test data set of 1000 participants, running the model, and then running the model on the rest of the data and comparing results. Goodness-of-fit tests on the models all indicated a good model fit for unadjusted and adjusted models. Collinearity for sociodemographic variables and CVD risk factors were not an issue. Participants with missing data for cognition scores were removed from the final data set used in analysis. To account for the loss of cognition data at follow-up (33.9% of the sample [8298 of 24 448]), we performed a sensitivity analysis using multiple imputation under the assumption of not missing at random. An additional sensitivity analysis was performed to exclude participants with any cognitive impairment at baseline (score of 6/6 on the SIS) and whose SIS score decreased by 2 or more points as ICI. Because reverse causation is a common concern in the association of perceived stress and cognition, we categorized perceived stress as no stress at both time points, resolved stress (only at first time point), new stress (only at second time point), and persistent stress (at both time points) and examined associations with ICI. All analyses were done using SAS, version 9.4 (SAS Institute Inc). All *P* values were from 2-sided tests and results were deemed statistically significant at *P* < .05.

## Results

The final analytic sample included 24 448 participants (14 646 women [59.9%] and 9797 men [40.1%]; median age, 64 years [range, 45-98 years]; 10 177 Black participants [41.6%] and 14 271 White participants [58.4%]) (Table 1). A total of 5589 participants (22.9%) reported having elevated levels of perceived stress. There were no major differences in clinical characteristics between included participants and those who were excluded. Participants with higher perceived stress (PSS score >5) were more likely to be younger, more likely to be female, and more likely to be Black. Participants with elevated levels of stress were also significantly less likely to have a college degree, had lower family income, and were living in the southeastern US states where rates of stroke are highest in the US (“stroke belt” and “stroke buckle” regions). Cardiovascular disease risk factors, such as hypertension, diabetes, and dyslipidemia, were also significantly more frequently observed among participants with elevated levels of perceived stress. Participants with elevated levels of stress were also less physically active, had a higher body mass index, and were more often current smokers.

**Table 1.** Descriptive Characteristics in the REGARDS Study Stratified by Baseline PSS Score

Characteristic	Participants, No. (%)			*P* value
	Total (N = 24 448)	Low stress (PSS score <5) (n = 18 859)	Elevated stress (PSS score ≥5) (n = 5589)	
Age, median (range), y	64 (45-98)	64 (45-83)	62 (45-93)	<.001
Sex				
Male	9797 (40.1)	8101 (43.0)	1696 (30.3)	<.001
Female	14 646 (59.9)	10 753 (57.0)	3893 (69.7)	
Race				
Black	10 177 (41.6)	7309 (38.8)	2868 (51.3)	<.001
White	14 271 (58.4)	11 550 (61.2)	2721 (48.7)	
Educational level				
College graduate	8502 (34.8)	7145 (37.9)	1357 (24.3)	<.001
Some college	6634 (27.1)	5170 (27.4)	1464 (26.2)	
High school graduate	6393 (26.1)	4706 (25.0)	1687 (30.2)	
<High school	2897 (11.8)	1820 (9.7)	1077 (19.3)	
Annual income, $				
<20 000	4323 (17.7)	2724 (14.4)	1599 (28.6)	<.001
20 000-34 000	5765 (23.6)	4350 (23.1)	1415 (25.3)	
35 000-74 000	7203 (29.5)	5895 (31.3)	1308 (23.4)	
>75 000	3952 (16.2)	3469 (18.4)	483 (8.6)	
Refused	3205 (13.1)	2421 (12.8)	784 (14.0)	
Region^a^				
“Stroke belt”	8572 (35.1)	6501 (34.5)	2071 (37.1)	<.001
“Stroke buckle”	5284 (21.6)	3993 (21.2)	1291 (23.1)	
Nonbelt	10 592 (43.3)	8365 (44.4)	2227 (39.8)	
CVD risk factors				
Hypertension	14 364 (58.8)	10 793 (57.2)	3571 (63.9)	<.001
Diabetes	5464 (22.3)	3918 (20.8)	1546 (27.7)	<.001
Dyslipidemia	13 745 (56.2)	10 581 (56.1)	3164 (56.6)	<.001
Health behaviors, No./total No. (%)				
Physical activity				
None	8526/24 091 (35.4)	6143/18 603 (33.0)	2383/5488 (43.4)	<.001
1-3 times per week	8710/24 091 (36.2)	6873/18 603 (36.9)	1837/5488 (33.5)	
≥4 times per week	6855/24 091 (28.5)	5587/18 603 (30.0)	1268/5488 (23.1)	
BMI				
Underweight	262/24 264 (1.1)	193/18 735 (1.0)	69/5529 (1.2)	<.001
Normal	5789/24 264 (23.9)	4525/18 735 (24.2)	1264/5529 (22.9)	
Overweight	8774/24 264 (36.2)	7040/18 735 (37.6)	1734/5529 (31.4)	
Obese	9439/24 264 (38.9)	6977/18 735 (37.2)	2462/5529 (44.5)	
Smoking				
Current	3549/24 354 (14.6)	2410/18 792 (12.8)	1139/5562 (20.5)	<.001
Past	9404/24 354 (38.6)	7487/18 792 (39.8)	1917/5562 (34.5)	
Never	11 401/24 354 (46.8)	8895/18 792 (47.3)	2506/5562 (45.1)	
Alcohol use				
Heavy	948/23 993 (4.0)	723/16 802 (4.3)	173/5484 (3.2)	<.001
Moderate	7954/23 993 (33.2)	5848/16 802 (34.8)	1538/5484 (28.0)	
None	15 091/23 993 (62.9)	10 231/16 802 (60.9)	3773/5484 (68.8)	

Abbreviations: BMI, body mass index; CVD, cardiovascular disease; PSS, Perceived Stress Scale; REGARDS, Reasons for Geographic and Racial Differences in Stroke.

^a^ The stroke belt and stroke buckle are the southeastern US states where rates of stroke are highest in the US. The stroke belt comprises North Carolina, South Carolina, Georgia, Tennessee, Mississippi, Alabama, Louisiana, and Arkansas. The stroke buckle comprises the coastal plains of North Carolina, South Carolina, and Georgia that have a higher stroke mortality rate than the remainder of the stroke belt.

When perceived stress was modeled as a continuous variable in the baseline data set, it was associated with 1.08 higher odds of prevalent cognitive impairment in the unadjusted model (OR, 1.08; 95% CI, 1.06-1.09) (Table 2). After adjusting for sociodemographic variables, CVD risk factors, lifestyle factors, and depressive symptoms, there was no appreciable change in the magnitude of the association (adjusted OR [AOR], 1.04; 95% CI, 1.03-1.06). Elevated levels of perceived stress (dichotomized as low stress vs elevated stress) were associated with 1.37 times higher odds of poor cognition after adjusting for sociodemographic variables, CVD risk factors, lifestyle factors, and depressive symptoms (AOR, 1.37; 95% CI, 1.22-1.53). After stratifying for sex and race in the full model, the following results were obtained: continuous PSS score ORs for both races and sexes were similar, with White men at 1.05 times (95% CI, 1.01-1.09) higher odds of prevalent cognitive impairment as the PSS score increased, White women at 1.04 times (95% CI, 1.00-1.09) higher odds, Black men at 1.05 times (95% CI, 1.01-1.09) higher odds, and Black women at 1.04 times (95% CI, 1.01-1.07) higher odds.

**Table 2.** Multivariable Odds Ratios for Association of PSS Score and Cognitive Impairment at Baseline (N = 24 448)

Model	Odds ratio (95% CI)	
	PSS score per unit change	PSS score dichotomized into low stress vs elevated stress
Unadjusted model	1.08 (1.06-1.09)	1.64 (1.48-1.82)
Adjusted model 1: sociodemographic variables including age, sex, educational level, race, and income	1.07 (1.05-1.09)	1.53 (1.37-1.70)
Adjusted model 2: model 1 and CVD risk factors including hypertension, diabetes, and hyperlipidemia	1.07 (1.05-1.08)	1.55 (1.39-1.72)
Adjusted model 3: model 2 and lifestyle factors including exercise, BMI, smoking, and alcohol use	1.07 (1.05-1.09)	1.57 (1.42-1.75)
Adjusted model 4: model 3 and depressive symptoms	1.04 (1.03-1.06)	1.37 (1.22-1.53)

Abbreviations: BMI, body mass index; CVD, cardiovascular disease; PSS, Perceived Stress Scale.

We explored the distribution of PSS scores stratified by cognitive status (normal vs impaired). Although there is a similar downward trend in the frequency of PSS scores, in the impaired cognitive status group, there was a larger percentage of people with higher PSS scores, suggesting that more people with higher levels of stress had impaired cognition (Figure).

**Figure.**
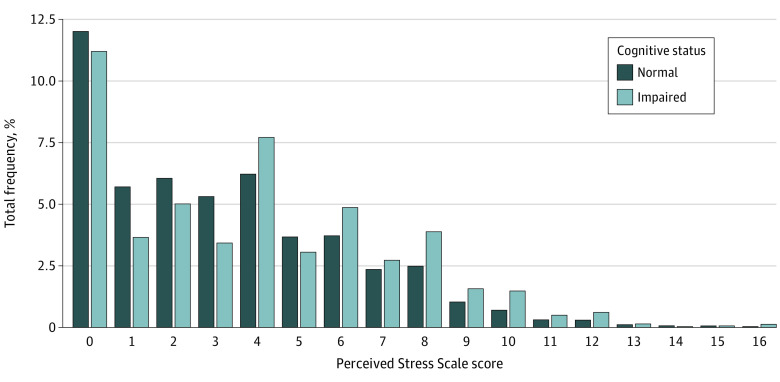
Distribution of Perceived Stress Scale Scores Stratified by Cognitive Status (N = 24 448)

We finally examined the association of PSS score with ICI. Only 16 150 participants had data on ICI. The association between baseline PSS score and ICI was significant after adjusting for sociodemographic variables and CVD risk factors (AOR, 1.22; 95% CI, 1.05-1.42) but was no longer significant after adjusting for sociodemographic variables, CVD risk factors, lifestyle factors, and depressive symptoms (AOR, 1.15; 95% CI, 0.98-1.36) (Table 3). When examining the association between change in PSS score (dichotomized into low stress vs elevated stress) from baseline to follow-up (until last observation) and ICI, the association was significant in the unadjusted model (OR, 1.62; 95% CI, 1.46-1.80) and after adjustment for sociodemographic variables, CVD risk factors, lifestyle factors, and depression (AOR, 1.39; 95% CI, 1.22-1.58). Dietary data were limited to 21 000 participants and, among this subset, additionally adjusting for nutrition did not alter our results. Our results with multiple imputation were similar to the analysis of complete records (ie, unadjusted OR, 1.35; 95% CI, 1.27-1.44; AOR, 1.19; 95% CI, 1.11-1.29). We also observed a clear dose response such that participants with persistent stress (AOR, 1.24; 95% CI, 0.95-1.63) or new stress (AOR, 1.16; 95% CI, 0.92-1.45) had more cognitive decline compared with those with resolved stress (AOR, 1.03; 95% CI, 0.81-1.32) or no stress (AOR, 0.81; 95% CI, 0.68-0.97). Our results also did not change in the sensitivity analysis when we used different cutoffs for cognitive impairment. When we examined the association of change in stress (modeled as a continuous variable with ICI at the most recent assessment), a change in perceived stress by 1 unit was associated with 1.04 higher odds of cognitive impairment after adjusting for sociodemographic variables, CVD risk factors, lifestyle factors, and depressive symptoms (AOR, 1.04; 95% CI, 1.02-1.06) (Table 3). For a 5-point difference, the AOR was 1.26 (95 CI, 1.10-1.33), which is comparable to the dichotomized results. There was no significant interaction between PSS score and any of age, race, or sex.

**Table 3.** Multivariable Odds Ratios for Association of PSS Score and of Change in Perceived Stress With Incident Cognitive Impairment at Most Recent Assessment (N = 16 150)

Model	Odds ratio (95% CI)		
	PSS score (low vs elevated stress from score at most recent follow-up)	Change in PSS score	
		Per unit change	Dichotomized into low vs elevated stress
Unadjusted model	1.19 (1.04-1.36)	1.07 (1.05-1.09)	1.62 (1.46-1.80)
Adjusted model 1: sociodemographic variables including age, sex, educational level, race, and income	1.27 (1.10-1.47)	1.03 (1.01-1.05)	1.41 (1.24-1.59)
Adjusted model 2: model 1 and CVD risk factors including hypertension, diabetes, and hyperlipidemia	1.22 (1.05-1.42)	1.03 (1.01-1.05)	1.38 (1.22-1.57)
Adjusted model 3: model 2 and lifestyle factors including exercise, BMI, smoking, and alcohol use	1.17 (1.00-1.36)	1.04 (1.02-1.06)	1.39 (1.22-1.58)
Adjusted model 4: model 3 and depressive symptoms	1.15 (0.98-1.36)	1.04 (1.02-1.06)^a^	1.39 (1.22-1.58)^b^

Abbreviations: BMI, body mass index; CVD, cardiovascular disease; PSS, Perceived Stress Scale.

^a^ *P* value for interaction terms in model 4 for continuous PSS score, change in perceived stress: age, *P* = .30; sex, *P* = .30; and race, *P* = .99.

^b^ *P* value for interaction terms in model 4 for dichotomized PSS score, change in perceived stress: age, *P* = .69; sex, *P* = .31; and race, *P* = .07.

## Discussion

Perceived stress is a common modifiable risk factor that is experienced by older individuals. Prior studies suggest that levels of stress increase with increasing age in a linear fashion.^32^ In this cohort from the REGARDS study, we investigated the association of perceived stress with cognition using longitudinal data. Participants with elevated levels of stress were more likely to have uncontrolled CVD risk factors and lifestyle factors (including physical inactivity, obesity, and smoking). Our results from this large cohort of Black and White individuals suggest that there is an independent association between perceived stress and cognition. The magnitude of the association did not meaningfully change after adjustment for sociodemographic variables, CVD risk factors, lifestyle factors, and depressive symptoms. In addition, using the longitudinal follow-up data in the REGARDS study, our results show that a change in perceived stress is also independently associated with ICI. The results were consistent whether perceived stress was continuous or categorical and across age, race, and sex.

Prior studies have shown that perceived stress is associated with lower cognitive scores and a faster rate of cognitive decline among elderly individuals.^33,34^ However, several of these studies had smaller sample sizes, were cross-sectional, and had lower representation from racial and ethnic minority communities. Aggarwal et al^6^ used data from the Chicago Health and Aging Project, which is from an urban setting in the Midwest. Similarly, the study from Katz et al^7^ used data from a single site (community in Bronx, New York) and had a smaller sample (n = 507). The REGARDS study is a nationally representative longitudinal study with a large sample size, and a high proportion of Black participants provides further evidence that perceived stress is associated with poor cognition and possibly precedes it. Other reports from the REGARDS study have shown the associations of perceived stress with incident coronary artery disease, all-cause mortality, and atrial fibrillation.^9,23^

Potential explanations of how perceived stress is associated with several unfavorable health outcomes include dysfunctional regulation of glucocorticoid secretion, alteration in autonomic tone, and an increased risk of unhealthy lifestyle behaviors.^35^ Multiple studies have also established the biological pathway, and there is evidence that elevated levels of stress biomarkers are associated with brain atrophy and possible cognitive decline.^36,37^ Furthermore, there is evidence that stress hormones, such as glucocorticoids, can cross the blood-brain barrier and may influence cognition.^37^ There is also evidence that experiencing a stressful event may affect the immediate level of cognition or the ability to perform cognitively challenging tasks.^38,39,40^ A study among middle-aged adults showed that people who reported greater job strain or stress about control of their occupation were at greater risk of cognitive impairment in later years.^41^ Individuals in different occupational roles may experience stress differently, and there are few studies that measure an individual’s subjective view of their own levels of stress.

In a few longitudinal studies, there is evidence to suggest that perceived stress and cognitive function are associated, but because of the differences in the measurement of stress and cognition, results have been difficult to compare.^6,16,42,43^ Stress-prone personality traits and negative life events have been associated with cognitive decline in previous cohort studies.^44,45^ The issue of racial differences is also of interest. Prior studies have shown that the association between proneness to distress and risk of Alzheimer disease is more substantial among White individuals than Black individuals. Compared with White individuals, Black individuals report greater exposure to chronic stressors, such as discrimination.^46^ In our study, Black participants reported higher levels of perceived stress, but the association between perceived stress and cognition did not differ by race. This finding suggests that high levels of perceived stress increase the risk of cognitive decline regardless of race. Understanding the association between perceived stress and cognitive functioning in different subgroups is important for designing targeted interventions and could have substantial public health implications.

### Strengths and Limitations

Our study has several strengths, including the large and diverse national sample, with oversampling of Black participants to allow for the examination of racial differences. Data collection used standardized questionnaires and anthropometric, cardiovascular, and health behavior parameters.

There are also some limitations to our study. Our measures of stress and depressive symptoms used validated but abbreviated versions of lengthier questionnaires. The participation rate in the REGARDS study was 49%. Although this rate is similar to other large national cohorts, it may limit generalizability of the findings. We had missing cognition data (34%) at follow-up, but our results were similar using validated multiple imputation methods. Despite adjustment of major confounders and longitudinal ascertainment of outcomes, unmeasured confounding could still potentially explain these associations. Finally, reverse causality can still explain some of our findings, although it is less likely given that our study is longitudinal and perceived stress was associated with ICI. There was also a clear dose response in the persistence of perceived stress and worse cognitive impairment. In addition, perceived stress was assessed at baseline among people without dementia and severe cognitive impairment, and thus we can still infer that perceived stress precedes poor cognition.

## Conclusions

This cohort study suggests that increased levels of perceived stress are associated with both prevalent cognitive impairment and ICI and that the association does not vary by age, race, and sex. Findings from our study could have important clinical applications, such as regular screening for stress among high-risk older adults when they present with cognitive decline in primary care. More research is needed to explore the underlying mechanisms for this observed association and to develop screening programs and targeted interventions to reduce stress among older adults at risk of cognitive impairment.

## Notes

Supplement.
Data Sharing Statement
