# Supplementary material for: Association of Stress With Cognitive Function Among Older Black and White US Adults
Source: JAMA Netw Open. 2023 Mar 7;6(3):e231860. doi: 10.1001/jamanetworkopen.2023.1860 (PMC9993177; doi:10.1001/jamanetworkopen.2023.1860)
Supplement: Supplement. — Data Sharing Statement [file jamanetwopen-e231860-s001.pdf]

Data Sharing Statement

Kulshreshtha. Association of Stress With Cognitive Function Among Older Black and White US Adults. *JAMA Netw Open*. Published March 07, 2023.
doi:10.1001/jamanetworkopen.2023.1860

Data
Data available: Yes
Data types: Deidentified participant data
How to access data: REGARDS Study website: https://www.uab.edu/soph/regardsstudy/
When available: With publication

Supporting Documents
Document types: Other (please specify)
Additional Information: Please email the study lead author for statistical code
How to access documents: Please email the study lead author for statistical code
When available: With publication

Additional Information
Who can access the data: Researchers whose proposed use of the data has been approved.
Types of analyses: For a specified purpose approved by the investigators of REGARDS cohort study
Mechanisms of data availability: Approval of a proposal by the investigators of REGARDS cohort study
Any additional restrictions: https://www.uab.edu/soph/regardsstudy/
